# GCP-Based Automated Fine Alignment Method for Improving the Accuracy of Coordinate Information on UAV Point Cloud Data

**DOI:** 10.3390/s22228735

**Published:** 2022-11-11

**Authors:** Yeongjun Choi, Suyeul Park, Seok Kim

**Affiliations:** 1 Department of Railroad Civil Engineering, Korea National University of Transportation, 157, Cheoldobangmulgwan-ro, Uiwang-si 16106, Korea; 2 Department of Railroad Convergence System, Korea National University of Transportation, 157, Cheoldobangmulgwan-ro, Uiwang-si 16106, Korea; 3 Department of Railroad Infrastructure System Engineering, Korea National University of Transportation, 157, Cheoldobangmulgwan-ro, Uiwang-si 16106, Korea

**Keywords:** point cloud, construction site, ground control point, object detection, unmanned aerial vehicles, coordinate accuracy, automatic systems

## Abstract

3D point cloud data (PCD) can accurately and efficiently capture the 3D geometric information of a target and exhibits significant potential for construction applications. Although one of the most common approaches for generating PCD is the use of unmanned aerial vehicles (UAV), UAV photogrammetry-based point clouds are erroneous. This study proposes a novel framework for automatically improving the coordinate accuracy of PCD. Image-based deep learning and PCD analysis methods are integrated into a framework that includes the following four phases: GCP (Ground Control Point) detection, GCP global coordinate extraction, transformation matrix estimation, and fine alignment. Two different experiments, as follows, were performed in the case study to validate the proposed framework: (1) experiments on the fine alignment performance of the developed framework, and (2) performance and run time comparison between the fine alignment framework and common registration algorithms such as ICP (Iterative Closest Points). The framework achieved millimeter-level accuracy for each axis. The run time was less than 30 s, which indicated the feasibility of the proposed framework.

## 1. Introduction

3D point cloud data (PCD) are a set of points with X, Y, and Z coordinates, and sometimes color information. These data points can accurately and efficiently capture the 3D geometric information of a target [1]. Recent advances in PCD capture technology and sensors have enabled cost-effective and accurate acquisition of PCD [2]. Owing to these advantages and the technical background, the construction industry has been acquiring construction site point clouds to enable efficient and safe project management [1,3]. The amount of research on the implementation of PCD in construction applications has increased in recent years, demonstrating the popularity of PCD in this domain [2]. The utilization of PCD exhibits high potential in civil and infrastructure applications, such as 3D reconstruction and construction automation [4,5]. Thus, many researchers have realized the potential of PCD, including construction quality control [6,7], progress monitoring [8,9], improving safety [3,10], and maintenance [11,12], by utilizing and analyzing PCD throughout the project lifecycle.

One of the most common approaches of generating PCD in remote sensing applications is the use of unmanned aerial vehicles (UAVs), which have become promising technologies in construction applications because they combine the advantages of aerial and terrestrial photogrammetry and provide distinct advantages, such as accessibility, flexibility, and low operating costs [13,14,15]. The most popular solution for generating PCD using UAV images is structure from motion (SfM) algorithm that extracts 3D structures (e.g., sparse point clouds) by identifying matching features from a set of 2D images acquired from multiple locations, orientations, and angles [15]. Owing to the advantages of SfM, which are simpler and more photo-processed than traditional photogrammetry, several researchers have combined UAVs with SfM in construction applications. For instance, Daftry et al. [16] proposed an SfM-based incremental 3D reconstruction framework with real-time computation for the accurate 3D facade reconstruction of buildings. Other studies have shown that UAV-based PCD are a competitive alternative to laser-scanning systems for complex as-built modeling [17].

However, UAV photogrammetry-based PCD can exhibit deformations or errors (e.g., inaccurate coordinates). Julge et al. [18] estimated that the errors of UAV-based PCD in road construction monitoring are two to three times the ground sampling distance (GSD); the flight altitude of a UAV significantly affects its accuracy [19]. In addition, poor weather conditions (e.g., cloudy conditions) degrade the UAV image quality, reducing the accuracy of the PCD [20]. The monotonous features of a substantial amount of vegetation in the flight area hinder the identification of matching features in the image, resulting in errors [21]. Bemis et al. [22] showed that an extended survey duration (over 30 min) resulted in changes in illumination (e.g., changing sun azimuth), significantly degrading accuracy. The factors causing errors, as mentioned in the above studies can occur during UAV surveys at construction sites. These errors sometimes prevent the PCD from meeting the accuracy requirements of certain construction applications. For instance, some applications such as broad geographic surveying do not require high PCD accuracy, but specific applications, such as displacement inspection, require high accuracy. In addition, improving the coordinate accuracy of PCD can efficiently contribute to construction-related tasks such as 3D location tracking of construction assets (e.g., workers and equipment) [23].

Therefore, several researchers have studied the factors influencing the accuracy of UAV-based PCD for implementing the PCD for construction applications [24]. Most of these factors include UAV survey plans, georeferencing techniques (e.g., optimal distribution and number of ground control points (GCPs)), UAV flight parameters (e.g., altitude and overlap settings of forward and side), and environmental affects [15,25,26,27]. In general, the achievement of sufficient accuracy of UAV-derived PCD for some construction-related applications is possible using GCPs, however, centimeter-level errors are always present [28]. In addition, the necessary multiple flights in huge areas, such as construction sites, are difficult owing to the battery limitations of UAVs. In this case, PCD registration algorithms are used to integrate the divided PCD, but the coordinate error often increases owing to the distinct limitations of each registration method [29,30]. Methodologies that improve the accuracy of the generated PCD are necessary; however, they have been studied by few researchers.

In construction-related applications, the most common method for achieving the best accuracy of UAV-derived PCD is the use of GCPs to match the key features of the PCD with known real-world coordinates (e.g., global navigation satellite systems (GNSS) coordinates) [31,32]. This requires obtaining GCPs in images collected from UAVs and manually selecting features, which is labor intensive. Therefore, software such as ArcGIS can automatically detect specific GCPs [33]. This can help expedite the imagery processing workflow. Automated methods are being realized mainly through a machine and deep learning and have high potential in the construction industry owing to their advantages, such as efficiency, process acceleration, and sustainability [34,35]. In particular, image-based deep learning has achieved promising results in the field of UAV remote sensing [36].

Recently, the improvement in the accuracy of UAV-generated PCD and their applications in construction have been receiving increasing interest. The use of automated methods to improve the accuracy of PCD can efficiently contribute to their effective construction applications. This study proposes a GCP-based automated framework to improve the coordinates accuracy of UAV-generated PCD. This study aims at developing a fast and accurate framework by integrating the promising image-based deep learning and PCD analysis methods and applying the proposed framework to construction sites. The proposed framework was tested and validated using UAV data collected from actual road construction sites. The remainder of this paper is organized as follows: Section 2 describes the details of the proposed framework; Section 3 introduces the first phase of the framework and the implementation of the GCPs detection model; in Section 4, the framework is validated using a case study; the contributions and future work of this study are discussed in Section 5.

## 2. Framework for Research Methodology

**Figure 1:**
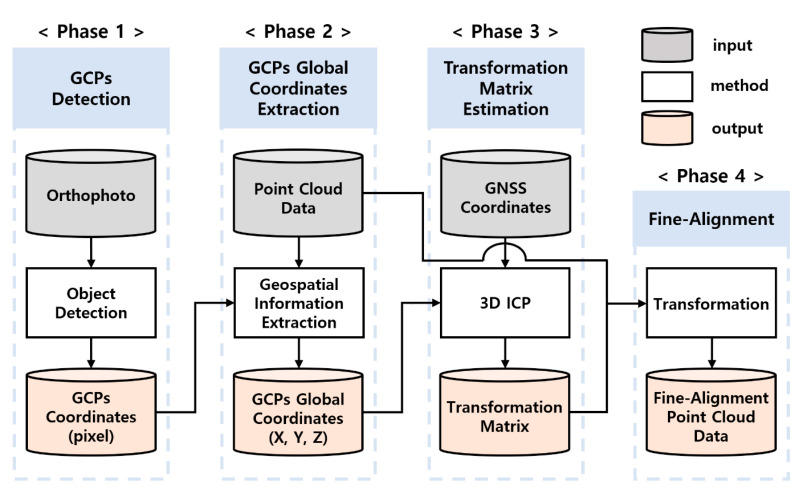
GCP-based automated fine alignment framework.

Figure 1 shows the automated framework of this study for the fine alignment of PCD. The framework comprises following four main phases: GCPs detection, GCPs global coordinate extraction, transformation matrix estimation, and fine alignment. First, a pre-trained detection model was used to detect GCPs in the orthophotos. The detection results provided 2D bounding box coordinates of the GCPs. Second, the global coordinates of the GCPs were extracted using their PCD and 2D coordinates. The longitude and latitude were extracted based on orthophoto information, and the Z-coordinates were calculated based on the location area of the GCPs in the PCD. The 3D iterative closest point (ICP) algorithm was then used to estimate the transformation matrix that minimizes the error between the GNSS coordinates and global coordinates of the GCPs. GNSS coordinates were measured and used by GNSS equipment at the site. Finally, fine alignment was performed based on the estimated transformation matrix. The main idea of this framework is to automatically improve coordinate information accuracy through PCD fine alignment, which incorporates multiple methods. The details of each phase of the framework are provided in the following sections.

### 2.1. Phase 1: GCPs Detection

The purpose of this phase is to detect the landmark where the GCPs are located from a UAV-generated orthophoto and obtain their location information (i.e., bounding box coordinates). Generally, the construction site has a huge working area; therefore, the orthophoto of the construction site acquired by the UAV commonly has a high resolution. To detect small GCPs from the orthophoto of the construction site, we select the YOLOv5 [37] model, which exhibits high accuracy and processing speed. Previous studies [38,39,40] have shown the possibility of object detection and the high potential of YOLOv5 for UAV imagery and orthophotos.

YOLOv5 is a state-of-the-art single-stage detector (SSD) implemented in PyTorch based on existing YOLO models. Specifically, the YOLOv5 model processes a raw image and proposes a confidence score and possible regions-of-interest (ROIs), which indicate the probability of the existence of an object. The YOLOv5 model includes following three main components: (1) Cross stage partial networks (CSPNet) [41], used as the backbone. CSPNet solves the problem of repetitive gradient information on a large-scale backbone and efficiently extracts the image features. In particular, CSPNet achieves significant benefits by reducing inference speed, improving accuracy, and reducing model size. (2) To boost the information flow, YOLOv5 uses a path aggregation network (PANet) [42] as a neck. PANet allows models to improve generalization accuracy through object scaling (e.g., GCP images from different altitudes) based on a feature pyramid network (FPN), which improves the propagation of low-level features in images. (3) Final detection is performed by the head of YOLOv5, and three different sizes are outputted from the layer to achieve multiscale prediction (e.g., small to large object detection). In general, GCPs are photographed in different sizes depending on the altitude of the position they are placed; therefore, multi-scale prediction potentiates GCPs detection. The detection results return the region bounding boxes of the GCP, and the pixel coordinates of the bounding boxes are used in the next process.

### 2.2. Phase 2: GCPs Global Coordinate Extraction

**Figure 2:**
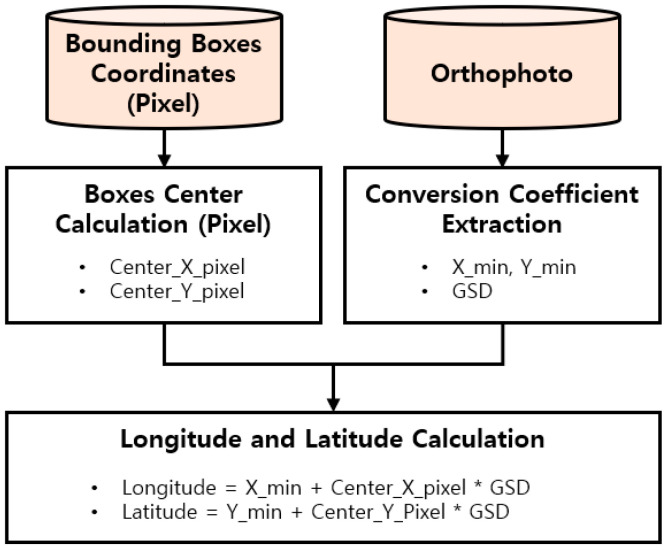
Conversion process of pixel coordinates to longitude-latitude coordinates.

This phase extracts the X, Y, and Z global coordinates of the detected GCP center using the GCP coordinates and PCD. Because the coordinates generated from the detected GCPs were pixel values, the X and Y coordinates were converted into longitude and latitude using geoinformation of orthophotos to obtain an accurate geographic location. Figure 2 shows the conversion process of the pixel coordinates to longitude–latitude coordinates. The conversion coefficients extracted from orthophotos include X_min, Y_min (e.g., corner coordinates), and GSD.

**Figure 3:**
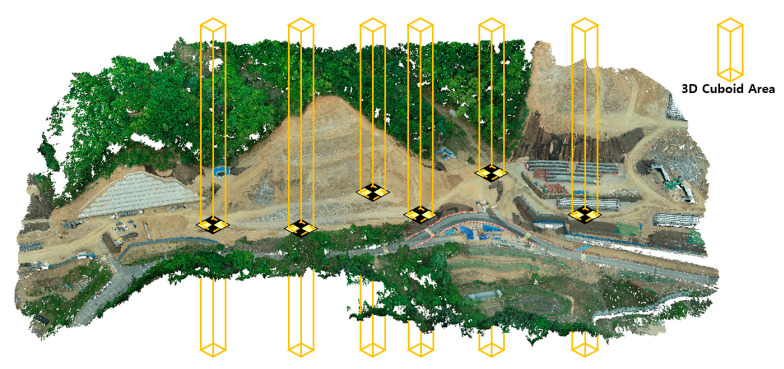
An idea for extracting Z-coordinates of GCPs from point cloud data.

The conversion results provide coordinate information of the location of GCPs in the PCD. Next, the Z-coordinates were calculated by integrating the coordinates of the point clouds within the GCP location area based on the conversion result. More specifically, the following steps were followed: (1) A 2D square area of a certain size having latitude–longitude coordinates as the center was set. (2) A 3D cuboid with the set 2D square as the top and bottom faces was generated. The height of the 3D cuboid was determined in a large range to include both the lowest and highest Z-values of all points. (3) The Z-coordinates of the GCPs were extracted by averaging the Z-coordinates of the point clouds in the cuboid area. The idea of extracting the Z-coordinates of the GCPs is shown in Figure 3. Although a square-shaped GCP is commonly used, their appearance depends on the their placement direction and viewpoint [43]. Therefore, the 2D square area needs careful setting to include only PCD within the GCP-located area. In this study, a minute difference between the centers of the bounding boxes and GCPs was confirmed, and the two coordinates were assumed to be the same.

### 2.3. Phase 3: Transformation Matrix Estimation

**Figure 4:**
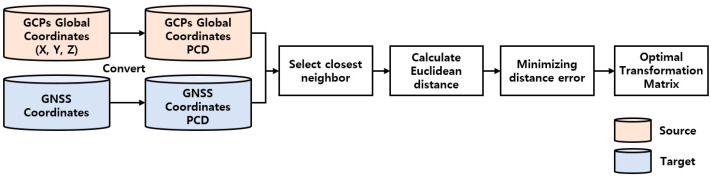
Performance sequence of transformation matrix estimation process.

The purpose of this process was to estimate the optimal transformation matrix by calculating the distance error between the extracted global coordinates of the GCPs and the coordinates measured by the GNSS equipment at the site. In other words, assuming that the GNSS coordinates were accurate, a matrix estimation process moved and rotated the global coordinates of the detected GCPs into GNSS coordinates. This process applied the 3D ICP [44] algorithm to estimate a transformation matrix that minimizes the error between two coordinate sets. Although ICP is a classic method, it has been continuously improved and has now become a comprehensive method [45]. The basic concept of ICP is still similar, and it has shown potential in the PCD of construction environments [30]. Figure 4 shows the performance sequence of this process, which involves the point-to-point ICP algorithm.

First, the GCPs global and GNSS coordinates were converted to PCD. The *k-*nearest neighbor search algorithm was then used to create a correspondence between the two-point sets. Finally, the optimal 4 × 4 transformation matrix was calculated by minimizing the sum of the squares of the Euclidean distances between the corresponding points. The object function that minimizes the distance between the corresponding points can be defined as

(1) $$\mathrm{Object}\;\mathrm{function}=\mathrm{minimize}\;\sum_{\left(p,q\right)\in K}\left||p-Tq|\right|,$$

where the $K=\left\{\left(p,q\right)\right\}$ are the correspondence sets from the target point cloud P (GNSS coordinates) and source point cloud Q (global coordinates of detected GCPs). T is the transformation matrix.

### 2.4. Phase 4: Fine Alignment

In this phase, fine alignment was performed by applying the transformation matrix estimated in Phase 3 to the PCD. The transformed PCD is calculated as

(2) $$\left[\begin{array}{c} x^{\prime }\\ y^{\prime }\\ z^{\prime }\\ 1 \end{array}\right]=T\left[\begin{array}{c} x\\ y\\ z\\ 1 \end{array}\right],\;T=\left[\begin{array}{cccc} r_{11} & r_{12} & r_{13} & t_{1}\\ r_{21} & r_{22} & r_{23} & t_{2}\\ r_{31} & r_{32} & r_{33} & t_{3}\\ 0 & 0 & 0 & 1 \end{array}\right],$$

where $\left(x^{\prime },\;y^{\prime },\;z^{\prime },\;1\right)$ is the transformed PCD and $\left(x,\;y,\;z,\;1\right)$ is the original PCD. T is the transformation matrix estimated in Section 2.3. The upper-left 3 × 3 submatrix of T ($r_{11},r_{12},r_{13},r_{21},\;r_{22},\;r_{23},r_{31},r_{32},r_{33}$) represents a rotation vector and the last column of T $\left(t_{1},t_{2},t_{3}\right)$ represents a translation vector. As a result of fine alignment, the PCD was moved and rotated to geometrically approximate the GNSS coordinates.

## 3. Implementation of GCPs Detection Model

This section describes the implementation of the GCPs detection phase. All computations of data processing, training, and validation were performed on a Windows 10 system support with an Intel(R) Core(TM) i7-10700KF CPU, 64 GB RAM, and NVIDIA GeForce RTX 2080Ti GPU. Specifically, the GCPs detection model, YOLOv5, was trained in Python 3.8.13.

### 3.1. UAV Images Aquisition

**Figure 5:**
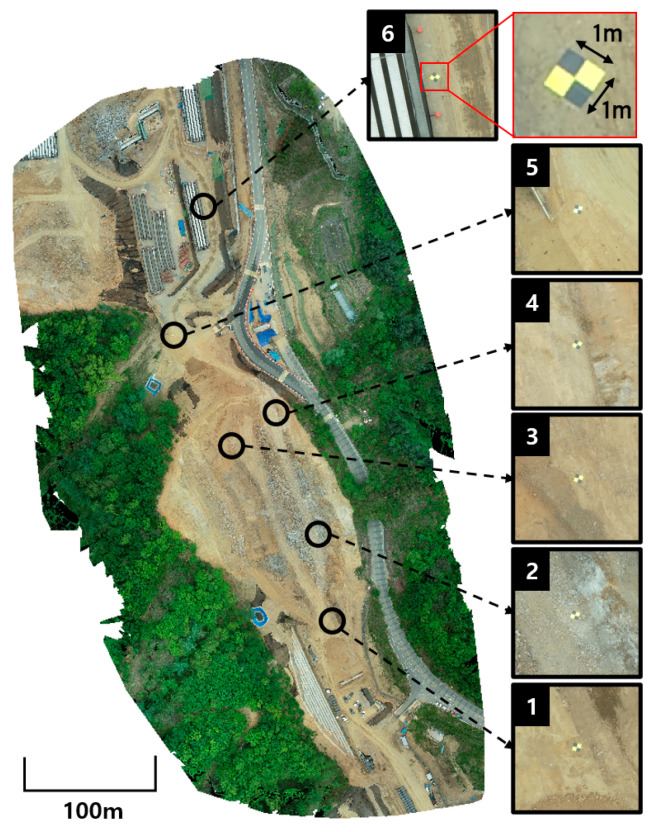
Entire flight area and placed GCPs.

The GCPs used in this study were manufactured in the form of 1 m × 1 m square considering the comprehensively used shape [43]. UAV flights were conducted twice at a road construction site near Gyeonggi-do, South Korea. The entire site area was approximately 200 m × 400 m, and six GCPs were placed throughout the area before the flight. The UAV hardware used for the flight was a DJI Phantom 4, and RGB images were acquired at approximately 100–110 m above ground using a DJI FC6310R camera composed of a CMOS sensor with 19.96 Mpix. As a result, 638 high-resolution images (5472 × 3648) were collected over two flights. The GCPs were small in the acquired images, occupying an average of 33 × 33 pixels in the image of 5472 × 3648 resolution, which was often poorly visible. Figure 5 shows the entire flight area and the six enlarged GCPs placed at the construction site.

### 3.2. Image Pre-Processing for Training

**Figure 6:**
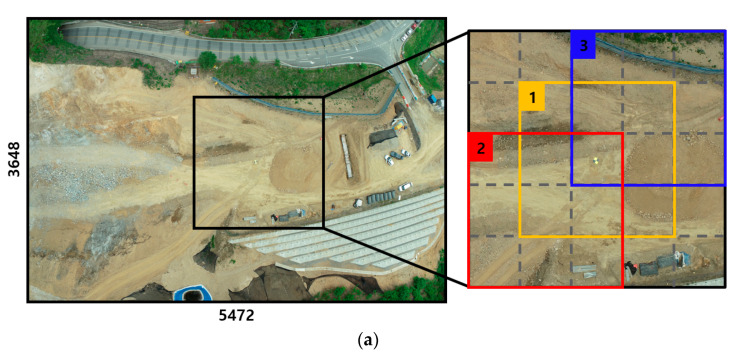

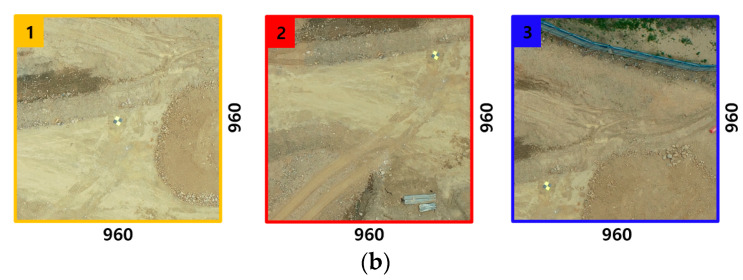
(**a**) Method of obtaining three tiles from one GCP in original (5372 × 3648) images for training, (**b**) Example of generated three tiles from one GCP.

For the training and validation of the GCPs detection model, sufficient images acquired by UAV were required. This study constructed a dataset using the acquired 638 images, with the following procedure. First, of the 638 images acquired, six images that do not contain GCPs and only terrain and vegetation were precluded in the dataset. Subsequently, 632 images were randomly split into training (70%), validation (20%), and test (10%) sets. The split of datasets was based solely on the number of acquired images; some images contained only one GCP, while others contained 2–4 GCPs. The average number of GCPs in one acquired image is 2.32. The number of GCPs included in the acquired image was small, and it is difficult to use the full size of the acquired image as a training input owing to memory limitations. Therefore, all images included in the sets were manually divided into 960 × 960 ‘tiles’ by determining the GCP placement area in the original (5372 × 3648) images. In addition, one GCP becomes three ‘tiles’ to enhance the number of training images. Each tile contained at least one GCP, and the GCP was distributed widely at not only the top and bottom, but also the center of the tile. Figure 6 shows the process of obtaining three tiles from one GCP in the acquired images and examples of tiles.

All generated tiles were manually annotated by using a web-based annotator, Roboflow. A team of two annotators carefully generates bounding boxes that enclose all GCPs within the tiles. All annotators double-checked all annotated images for quality control of the dataset. As a result of the annotation, 3197 training tiles, 756 validation tiles, and 382 test tiles were generated. In addition, image augmentation methods were used to train a robust detection model. Augmentation enhances the size and quality of training datasets and helps to train a robust model for variate environmental conditions, UAV altitudes and tilt, and different landscapes [46]. In this study, we chose image augmentation methods based on the characteristics of the GCPs. For instance, a square-shaped GCP has the same shape even when rotated by 90°; therefore, the 90° rotation method was not considered. Besides, the color information of GCP may be different owing to changes in environmental conditions (e.g., bright or cloudy days); therefore, a color space adjustment technique was used. In conclusion, image saturation (between −50% and 50%), brightness (between −35% and 35%), exposure (between −30% and 30%), blur (between 1 and 3 pixels), noise (between 1% and 20%), and rotation (between 1° and 40°) methods were used for image augmentation. The degree of each augmentation method was randomly selected from the range in parentheses. Table 1 lists the number of images and tiles per training, validation, and test sets.

The detection model developed in this study should detect GCPs in high-resolution orthophotos acquired at construction sites. However, GCPs Detection of the images with significantly high resolution may not be possible because of GPU memory limitations. Therefore, we chose to resize the resolution of all datasets from 960 × 960 to 480 × 480 pixels. A model trained with resized images can perform GCPs detection on orthophotos with a GSD of approximately 6 cm. For instance, if there is a 10,000 × 10,000 resolution orthophoto containing a 33 × 33 pixel GCP, YOLOv5 trained with 16.5 × 16.5 pixels GCP can perform GCPs detection by resizing the orthophoto to 5000 × 5000 resolution. This approach is partially free from GPU memory limitations when performing detection.

### 3.3. Training and Evaluation

The training set consisted of 22,379 images with 480 × 480 pixels, including the augmented tiles. The training and validation sets were used in the process of training the detection model, whereas the test set was only used for the independent evaluation of the model performance. Among the available YOLOv5 architectures, this study selected YOLOv5L because of its better performance over a smaller architecture (i.e., YOLOv5s). Model training was performed by transfer learning of pretrained weights from a COCO dataset [47]. The model was trained for 50 epochs and was interrupted after 50 consecutive epochs. The batch size was set to 16, which was the maximum possible size within the constraints of the hardware used, and the image resolution was set to 480 × 480 pixels.

The model was evaluated in terms of precision, recall, and mean average precision (mAP). The mAP corresponds to intersection over union (IoU) thresholds of 0.5 (mAP@0.5), and different IoU thresholds range from 0.5 to 0.95 (mAP@[0.5:0.95]). The IoU was defined as the ratio of the intersection area to the union area of the two bounding boxes. To calculate precision and recall, the true positives $\left(TP\right)$, false positives $\left(FP\right)$, and false negatives $\left(FN\right)$ must be computed (Equations (3) and (4)). In this study, a combination of predicted and annotated boxes was considered a true positive when the IoU was equal to or greater than a certain threshold (0.2 in this study).

(3) $$\;Precision=\frac{TP}{TP+FP}$$

(4) $$Recall=\frac{TP}{TP+FN}$$

where *TP* is the number of true positives, *FP* is the number of false positives, and *FN* is the number of false negatives.

**Figure 7:**
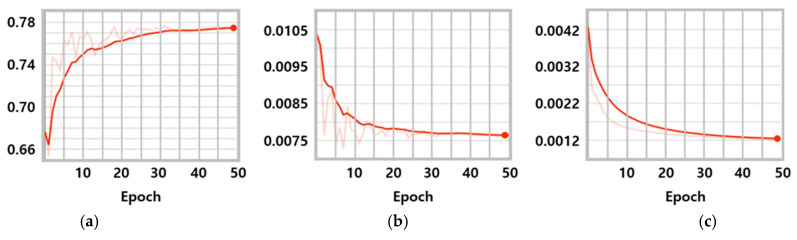
Training results obtained on the validation set for 50 epochs, (**a**) mAP@[0.5:0.95] curve; (**b**) curve of box loss; (**c**) curve of objectness loss.

The overall training time was 4 h 59 min, and training was interrupted after 50 consecutive epochs. The model with the highest mAP@[0.5:0.95] was found at the 50th epoch (Figure 7).

**Figure 8:**
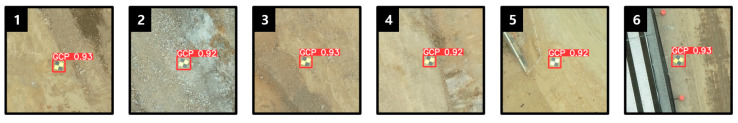
Detection results of six GCPs in orthophoto.

The trained model achieved a 0.7749 mAP@[0.5:0.95] on the validation set. The mAP@0.5 reached its maximum at the beginning of the training. The box loss represents how well the model can find the center of the GCP and how well the predicted bounding box covers the GCP (0.0076 in this study). The objectness represents the probability of the existence of a GCP in the proposed ROI. If a predicted bounding box is not assigned to a GCP, an objectness loss occurs (0.0012 in this study). The best-performing model for the validation set was applied to the test set and orthophotos. Specifically, the orthophoto was created using Pix4D software based on the overall acquired images and had a resolution of 10,019 × 16,108. Figure 8 shows an example of the GCPs detection results for an orthophoto. The trained model demonstrated the ability to perform detection even on a high-resolution orthophoto.

## 4. Case Study

A case study was conducted to validate the proposed fine alignment framework. Data were collected from three flights in the case study area and two different experiments were performed: (1) experiments on the fine alignment performance of the developed framework and (2) performance and run time comparison between the fine alignment framework and common registration algorithms. Owing to the battery limitations of UAVs, flight over huge construction areas using a single battery may not be possible. Therefore, multiple flights are often conducted with battery changes for the entire area survey. In this case, it is necessary to register the divided site PCD to generate an integrated site PCD. ICP is the most common method for fine registration and was therefore chosen for performance comparison [48]. Thus, this case study confirms both the accuracy and site applicability of the framework.

### 4.1. UAV Data Acquisition and Pre-Processing

For this case study, we collected UAV data from an actual construction site near Cheongju-si, South Korea. Being a road construction site, the earthwork area is horizontally long, and vegetation is distributed above and below the area. The entire site area was approximately 900 m × 350 m, and five 1 m × 1 m GCPs were placed throughout the study area before the UAV flight. The coordinates of the GCPs were determined using the GS18i GNSS equipment (Table 2).

**Figure 9:**
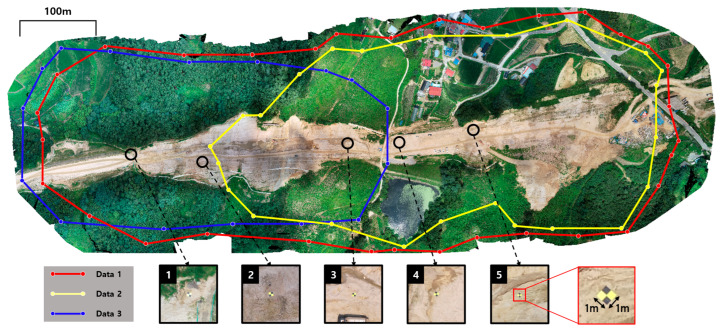
The whole area of the case study. Three flights were conducted: (1) red polyline indicates first flight area, called Data 1; (2) yellow polyline indicates second flight area, called Data 2; (3) blue polyline indicates third flight area, called Data 3.

The UAV flights were conducted three times to perform two different experiments (Figure 9). The UAV hardware used for the flight was a DJI Phantom 4 RTK, and the equipped camera specifications were the same as those in Section 3.1. All flights took place approximately 100–120 m altitude above the ground level. Orthophotos and PCD were then generated for each flight using Pix4D software (Data 1, 2 and, 3 in Figure 9). More specifically, orthophotos were exported, and Data1 had a native resolution of 27,832 × 10,743 and a GSD of 3.27 cm, Data 2 had a resolution of 21,344 × 10,213 and a GSD of 3.34 cm, and Data 3 had a resolution of 20,546 × 10,330 and GSD of 3.08 cm. Data 1, 2, and 3 were all used for the performance experiments of the framework, and Data 2 and 3 were used for the performance comparison between the framework and registration algorithms.

### 4.2. Experimental Method

GCPs detection was performed by resizing each of the three orthophotos so that the GCP occupied 16.5 × 16.5 pixels (see Section 3.2). The 2D square area for extracting the Z-coordinate of the detected GCPs was set to 0.7071 m considering the shape of the 1 m × 1 m GCP, and the height of the 3D cuboid was set to 200 m vertically based on 0 (see Section 2.2). The metric used to evaluate the performance of PCD fine alignment was the root-mean-square error (RMSE). The first experiment showed the RMSE between the detected GCPs and GNSS coordinates, and showed the decrease in the RMSE between the two coordinates after fine alignment. The second experiment used registration algorithms between PCD pairs of Data 2 and 3. Because the overlapping area between the two datasets was small, each PCD was cut to increase the overlap ratio. As a result, point-to-point ICP and plane-matching methods were applied to the data with an overlap ratio of approximately 70%. The GCPs coordinates obtained from the PCD of the registration result were compared with the GNSS coordinates using the RMSE. Finally, we compared the runtime of the proposed framework and registration algorithms and evaluated its applicability to the site.

### 4.3. Experimental Results

**Figure 10:**
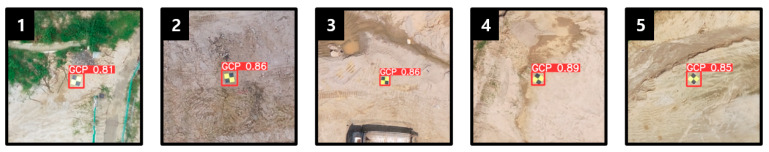
Detection results of five GCPs in orthophoto of Data 1.

Figure 10 shows an example of the GCPs detection results for an orthophoto of Data 1. The model correctly detected only GCPs with a confidence score of 0.6, and there was no detection error. The proposed framework effectively detects GCPs in high-resolution orthophotos of construction environments.

**Figure 11:**
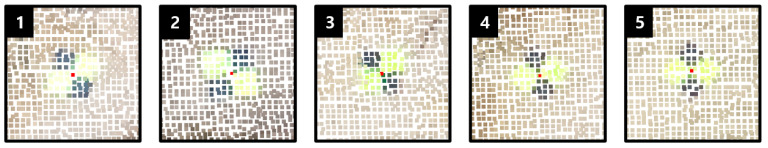
Visualization of the fine alignment results of Data 1. Red points represent GNSS coordinates.

Table 3 presents the quantitative experimental results of the fine alignment performance of the developed framework. Each of the values in Table 3 represents the difference in the distance from the GNSS coordinates. As a result of fine alignment, the RMSEs of Data 1, 2, and 3 were reduced from 0.0666 m to 0.0333 m, from 0.1126 m to 0.0384 m, and from 0.0708 m to 0.0128 m, respectively. This implies that the fine alignment method improved the coordinate information accuracy of the PCD by 0.0333, 0.0742, and 0.058 m, respectively. Most of the individual axis accuracies were improved. Owing to the characteristics of the fine alignment objective function, in rare cases, the accuracy of the individual axes may decrease, but the overall accuracy is improved. In Data 3, notably, the framework achieved millimeter-level accuracy for each axis and 1 cm-level RMSE accuracy. The results indicate that the proposed method can effectively improve the PCD accuracy. A visualization of the fine alignment results is shown in Figure 11. The red points represent GNSS coordinates.

In comparative analysis with the results of Pix4D, an additional effect of the developed framework was observed. The RMSEs of Data 1, 2, and 3 according to the Pix4D quality report were 0.042, 0.028, and 0.029, respectively. This implies that compared to Pix4D, the fine alignment accuracy is increased by 0.009 m, −0.0104 m, and 0.01 6m, respectively. Note that several trials and errors were required to obtain the optimal output of Pix4D considering the site characteristics, such as dense vegetation like Figure 9. Therefore, for the additional experiment, we generated new Data 1 without georeferencing and parameter optimization. The difference between the new Data 1 and the GNSS coordinates is about several meters because it was not georeferenced. Subsequently, a fine alignment experiment was performed on the new Data 1. The difference of several meters has been reduced to 0.0424 m as a result of fine alignment. Depending on the user’s proficiency, the Pix4D georeferencing and parameter optimization process can require significant effort. The fine alignment result indicates that labor and time to achieve improved outputs such as trial-and-error and georeferencing processes can be reduced by a developed framework.

**Figure 12:**
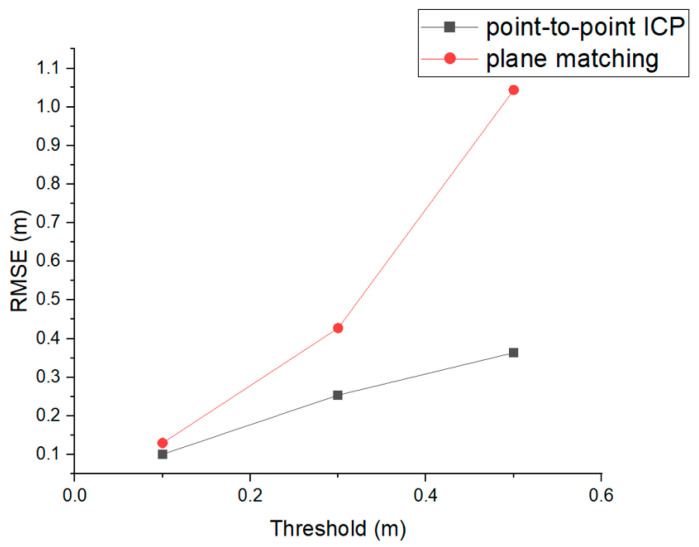
Registration RMSE of two algorithms according to the thresholds of 0.1, 0.3, and 0.5.

In addition to the performance of the proposed method, considerable site applicability was observed in additional experiments. This case study comparatively analyzed the performance of fine alignment and two common registration algorithms, that is, point-to-point ICP and plane-matching algorithms. Both registration algorithms are implemented in open3D, an open-source library [49]. Experiments were performed using registration thresholds of 0.1, 0.3, and 0.5 between Data 2 and 3 pairs. This means that within distances of 0.1, 0.3, and 0.5 m, the algorithm found a correspondence between two sets of points. Figure 12 shows the registration RMSE of the two algorithms according to the threshold. This shows that the RMSE increased as the threshold increased. When the threshold was 0.1, the RMSE of point-to-point ICP was 0.1005 m and the RMSE of plane matching was 0.1297 m. In comparison, fine alignment achieved 0.0384 m and 0.0128 m in Data 2 and 3, respectively, which means that higher accuracy can be achieved than with common registration algorithms.

Additionally, the runtimes of the proposed framework and the above two registration algorithms were compared. The results are presented in Table 4. Table 4 means that the run time of the developed framework is faster than the plane matching regardless of the threshold, and also faster than point-to-point ICP when the ICP threshold is over 0.5. Note that because Data 2 and 3 were cut before registration, point-to-point ICP and plane matching resulted in a slightly faster time than when applied to the original data. In addition, determining the optimal registration threshold requires trial and error. The runtime of the framework applied to all data was less than 30 s. More specifically, approximately 70% of the total run time was spent in Phase 1 and 10% each in Phases 2, 3, and 4. The results of the first and second experiments show that the proposed framework is applicable to the construction site and increases the accuracy of the PCD; the run time is also fast.

## 5. Summary, Contributions and Future Work

### 5.1. Summary

This study presents a GCP-based fully automated framework for improving the accuracy of coordinate information on UAV point clouds. The proposed framework comprises four phases. In Phase 1, the detection model detected all GCPs in the orthophoto. Phase 2 involved the extraction of the 3D global coordinates of the GCP based on the detection results and PCD. Phase 3 estimated a transformation matrix for fine alignment based on the GNSS coordinates and global coordinates of the GCPs. Finally, the PCD was fine aligned using the estimated matrix. The accuracy and runtime of the proposed framework were tested using UAV data acquired from the construction site. The results showed that improving the accuracy of PCD using the proposed framework is effective, fast, and feasible.

### 5.2. Contributions

The contributions of this study are as follows: First, this study proposed a novel post-processing method to improve the errors resulting from image-based 3D structure estimation (e.g., structure from motion). The framework achieved mm-level accuracy for each axis and 1cm-level RMSE accuracy and can contribute to improving the accuracy of additional analysis tasks (e.g., 3D location tracking of construction assets and earthwork volume estimation). Second, vision-based methods and 3D information extraction methods were integrated to automatically improve PCD accuracy. The proposed framework works within seconds without human intervention; therefore, it is easy to integrate into the data acquisition framework and has advantages in terms of automation. Finally, the proposed framework showed the potential to achieve consistent accuracy without parameter optimization and georeferencing. Experimental results on data generated without georeferencing and parameter optimization supported this possibility.

### 5.3. Limitations and Future Work

However, several research challenges remain unaddressed. The performance of the proposed framework depends mainly on the accuracy of the GCPs detection model. The center of the detected bounding box may not always be the exact center of the object. Although these accuracy issues are often observed in vision-based deep-learning approaches, future studies can investigate and replace improved vision-based models. In addition, pixel-level image-processing methodologies may be incorporated into the detection model. The solution for the future is to detect GCPs directly in 3D PCD and identify the centroid. Although processing PCD with learning-based methods is still challenging, due to the inherent specifications of PCD such as unorderdness, irregularity, and unstructured, this approach can simplify the proposed framework [50].

## Figures and Tables

**Table 1:** Number of images, tiles, and augmentation results per training, validation, and test sets.

Dataset	Images	Tiles	Augmentation	
Training	446	3197	Saturation	3197
			Brightness	3197
			Exposure	3197
			Blur	3197
			Noise	3197
			Rotation	3197
Validation	125	756	N.A.	
Test	61	382	N.A.	

**Table 2:** GCPs coordinates of the case study area measured by GNSS equipment.

GCP Number	X Coordinates	Y Coordinates	Z Coordinates
1	252,124.345	463,707.495	96.914
2	252,228.311	463,692.878	105.361
3	252,476.020	463,722.543	102.988
4	252,534.588	463,724.533	98.485
5	252,654.625	463,744.767	96.595

**Table 3:** Quantitative results on the fine alignment performance of the developed framework. Each value means the difference from the GNSS coordinates.

GCP Number	Data 1					
	GCPs Detection Results			Fine Alignment Results		
	X	Y	Z	X	Y	Z
1	0.0240	0.0508	0.0606	0.0019	0.0285	0.0072
2	0.0217	0.0954	0.0043	0.0038	0.0379	0.0153
3	0.0150	0.0271	0.0198	0.0380	0.0089	0.0221
4	0.0635	0.0436	0.0244	0.0397	0.0073	0.0122
5	0.0262	0.0191	0.0124	0.0040	0.0078	0.0018
Average RMSE(m)	0.0666			0.0333		
GCP number	Data 2					
	GCPs detection results			Fine alignment results		
	X	Y	Z	X	Y	Z
3	0.0284	0.0616	0.1562	0.0397	0.0197	0.0126
4	0.0458	0.0284	0.0407	0.0460	0.0290	0.0174
5	0.0162	0.0689	0.0707	0.0062	0.0092	0.0048
Average RMSE(m)	0.1126			0.0384		
GCP number	Data 3					
	GCPs detection results			Fine alignment results		
	X	Y	Z	X	Y	Z
1	0.0049	0.0869	0.0032	0.0063	0.0115	0.0042
2	0.0105	0.0181	0.0259	0.0018	0.0162	0.0058
3	0.0170	0.0173	0.0888	0.0045	0.0050	0.0016
Average RMSE(m)	0.0708			0.0128		

**Table 4:** Run time of the proposed framework and two registration algorithms.

		Data 2	Data 3
Fine alignment framework		21.8 s	18.61 s
point-to-point ICP	Threshold 0.1	29.35 s	
	Threshold 0.3	34.95 s	
	Threshold 0.5	39.16 s	
Plane matching	Threshold 0.1	64.23 s	
	Threshold 0.3	68.32 s	
	Threshold 0.5	73.15 s	

## Data Availability

Not applicable.
